# Heterogeneous chromosome 12p deletion is an independent adverse prognostic factor and resistant to bortezomib-based therapy in multiple myeloma

**DOI:** 10.18632/oncotarget.3319

**Published:** 2015-03-20

**Authors:** Fei Li, Yan Xu, Ping Deng, Ye Yang, Weiwei Sui, Fengyan Jin, Mu Hao, Zengjun Li, Meirong Zang, Dehui Zhou, Zhimin Gu, Kun Ru, Jianxiang Wang, Tao Cheng, Lugui Qiu

**Affiliations:** ^1^ State Key Laboratory of Experimental Hematology, Institute of Hematology and Blood Disease Hospital, Chinese Academy of Medical Sciences and Peking Union Medical College, Tianjin 300020, China; ^2^ Department of Hematology, The First Affiliated Hospital of Nanchang University, Nanchang 330006, China; ^3^ Umbilical Cord Blood Bank of Tianjin, Tianjin 300020, China; ^4^ Department of Science and Education, The First Affiliated Hospital of Nanchang University, Nanchang 330006, China; ^5^ Department of Internal Medicine, University of Iowa Carver College of Medicine, Iowa City, IA 52246, USA; ^6^ Tumor Center, The First Hospital of Jilin University, Changchun 130021, China

**Keywords:** 12p13 deletion, *CD27* gene, prognosis, bortezomib, multiple myeloma

## Abstract

The deletion of 12p (del(12p)) has been described as a novel negative prognostic marker in multiple myeloma (MM) and has gained increasing attention in recent years. However, its impact on MM is still controversial. In this study, we comprehensively evaluated the clinical impact of 12p13 deletion using fluorescence in situ hybridization (FISH) on 275 newly diagnosed MM cases treated in a prospective, non-randomized clinical trial (BDH 2008/02). The results showed that deletion of 12p13 was detected in 10.5% of newly diagnosed cases and associated with multiple indicators for high tumor burden including ISS III, BM plasmacytosis larger than 50%, and renal lesion. Moreover, the cases with 12p13 deletion typically had higher incidence of del(17p), IGH translocation and t(4;14). Patients with del(12p) conferred significantly adverse prognosis for PFS and OS, even in patients subjected to bortezomib-based therapy. When adjusted to the established prognostic variables including del(13q), del(17p), t(4;14), amp(1q21), ISS stage and LDH, del(12p13) remained the powerful independent adverse factor for PFS (*P* = 0.007) and OS (*P* = 0.032). In addition, del(12p13) combined with high β2-MG, high LDH and bone lesion can further identify subpopulations with high-risk features. Our results strongly supported that del(12p13) can be used as a valuable prognostic marker in MM.

## INTRODUCTION

Multiple myeloma (MM), a neoplasm of plasma cells, is characterized by complex chromosomal abnormalities. The cytogenetic abnormalities are the hallmark of MM and commonly used as the clinical predictors for determining the stage of disease and providing the guidance for therapeutic strategies [1]. Risk-stratification system based on genetic indicators has been established and recommended by mayo clinic and International Myeloma Working Group (IMWG) in recent years [2, 3]. Routine evaluation factors consist of deletion of 17p (del(17p)), t(4;14) and t(14;16) detected by fluorescence in situ hybridization (FISH) [4], however, none of these factors can completely explain the heterogeneity in this disease. New techniques such as single-nucleotide polymorphism (SNP)-based mapping array and array comparative genomic hybridization (aCGH) can provide a deeper knowledge of the diversity and heterogeneity of cytogenetic abnormalities in MM [5, 6]. So, the question of identifying some noval prognostic factors to better risk stratify patients in the management of myeloma is becoming an important issue.

Chromosome 12p deletion has been recently reported to exist in approximately 10% of MM patients and reveals poor prognosis [7, 8]. However, the practical prognostic value of del(12p) in MM remains controversial. Although it has been identified by using FISH in several studies [5], the failed confirmation is still observed in other series of studies [9, 10]. Moreover, the status of 12p aberration in MM and other plasma cell disorders are still unclear.

*CD27* gene, a member of the tumor necrosis factor receptor (TNFR) family, is regarded as a putative disease related gene located in 12p13.31. Zhan et al. have disclosed that the low expression of *CD27* could predict high-risk value by gene expression profiling (GEP) in MM [11]. Other studies have also confirmed this viewpoint by flow cytometry and immunohistochemical analysis [12, 13]. In order to explore the status of 12p deletion in MM and other plasma cell disorders and its prognostic value, a cohort of 275 patients with newly diagnosed MM from a prospective, non-randomized clinical trial (BDH 2008/02) has been analyzed by detection 12p13.31 using FISH in this study. Similarly, in order to compare the incidence of 12p aberration among different stages of plasma cell dyscrasias, the patients including 90 relapsed MM, 8 secondary plasma cell leukemia (sPCL) and 7 monoclonal gammopathy of undetermined significance (MGUS) were enrolled.

## RESULTS

### Patient's characteristics

A total of 275 newly diagnosed MM patients were subjected to the detection of 12p13 deletion. The median age of the patients was 58 years old (range, 26–83 yr) with the median follow-up time of 36 months from the diagnosis. Patients including 90-relapsed MM, 8 sPCL and 7 MGUS were enrolled for the analysis to compare the incidence of 12p aberration among different stages of plasma cell dyscrasias. The clinical characteristics of 275 patients in arm A and arm B were shown in Table 1. There was no significant difference in clinical and cytogenetic characteristics between both groups.

**Table 1:** The characteristics of 275 newly diagnosed MM patients

Characteristics	Arm A *N* = 138	Arm B *N* = 137	*P*
Age (years), median (range)	58 (26–83)	58 (30–81)	0.286
Sex, F/M	85/53	86/51	0.963
Isotype, N (%)			
IgG	61/130 (46.9)	71/135 (52.6)	0.441
IgA	34/130 (26.2)	27/135 (20.0)	
IgD	3/130 (2.3)	9/135 (6.7)	
IgM	1/130 (0.8)	1/135 (0.7)	
Light chains	26/130 (20.0)	22/135 (16.3)	
Non-secretory	5/130 (3.8)	5/135 (3.7)	
ISS stage, N (%)			0.656
I	22/132 (16.7)	18/131 (13.7)	
II	45/132 (34.1)	51/131 (38.9)	
III	65/132 (49.2)	62/131 (47.3)	
β2-microglobulin (mg/dL)	5.1 (1.0–27.2)	5.1 (1.0–40.0)	0.913
Durie-Salmon stage, N (%)			0.337
I–II	16/134 (11.9)	21/131 (16.0)	
III	118/134 (88.1)	110/131 (84.0)	
BM plasmacytosis (%), median (range)	14.5 (5–58)	30 (3–90)	0.591
Renal lesion, N (%)			0.433
None	113/138 (81.9)	107/137 (78.1)	
Present	25/138 (18.1)	30/137 (21.9)	
Bone lesions, N (%)			0.438
None	39/138 (28.3)	32/137 (23.4)	
Present	99/138 (71.7)	105/137 (76.6)	
Cytogenetic abnormalities, N (%)			
Del(13q)	52/138 (37.7)	59/137 (43.1)	0.363
Del(17p)	14/126 (11.1)	17/129 (13.2)	0.614
IGH translocation	68/119 (57.1)	72/124 (58.1)	0.884
t(11;14)	15/102 (14.7)	21/113 (18.6)	0.447
t(4;14)	19/102 (18.6)	26/113 (23.0)	0.370
t(14;16)	4/102 (3.9)	5/113 (4.4)	0.854
Amp(1q21)	54/111 (48.6)	57/119 (47.9)	0.910
High-risk [(any t(4;14), t(14;16) or del(17p)]	44/128 (34.4)	43/128 (33.6)	0.895

F, female; M, male; ISS, International Staging System; Ig, immunoglobulin; BM, bone marrow

### Chromosome 12p13 aberration in plasma cell dyscrasias

**Figure 1:**
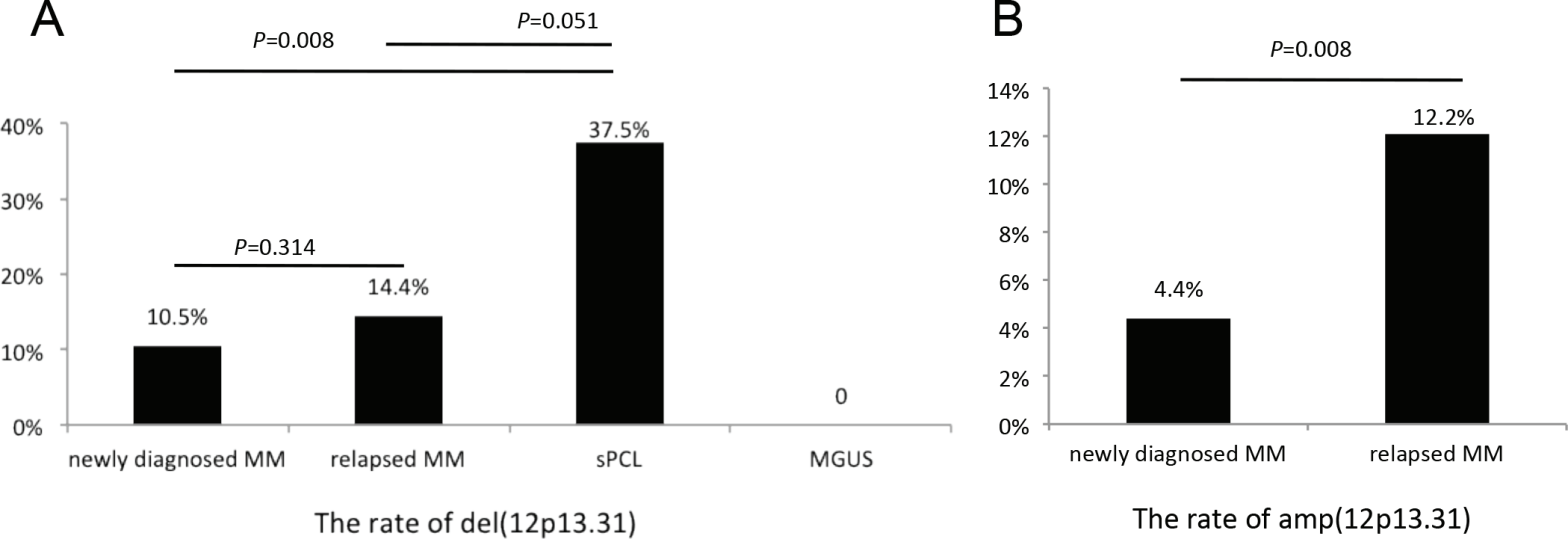
The deletion (A) and amplification (B) rates in plasma cell dyscrasias

In this series of 380 patients, the deletion of 12p13 was detected in 29 (10.5%) of 275 newly diagnosed and 13 (14.4%) of 90 relapsed patients (*P* = 0.314). Moreover, in patients with sPCL, 37.5% (3/8) of patients with 12p13 deletion were detected and revealed higher deletion rate than newly diagnosed and relapsed patients (*P* = 0.008, 0.051). However, none of 7 MGUS patients had this deletion. Interestingly, we also found that 4.4% (12/275) of newly diagnosed and 12.2% (11/90) of relapsed patients had 12p13 gain and revealed the higher gain rate in relapsed patients (*P* = 0.008). The comparison of deletion/gain rate in patients with plasma cell dyscrasias was shown in Figure 1A and 1B.

### The deletion of 12p13 is associated with multiple factors for high tumor burden and negative outcome in newly diagnosed MM

Clinical factors and genetic abnormalities associated with del(12p) were shown in Table 2. From these data, we found that del(12p) was significantly correlated with many factors for high tumor burden including ISS III (*P* = 0.006), BM plasmacytosis larger than 50% (*P* = 0.034), and renal lesion (*P* = 0.039). Moreover, cases with 12p13 deletion typically had a higher incidence of del(17p) (39.3% vs. 8.8%, *P* < 0.001), IGH translocation (75.0% vs. 55.3%, *P* = 0.048) and t(4;14) (38.5% vs. 18.5%, *P* = 0.019). We defined patients with the abnormalities of t(4;14), t(14;16) or del(17p) as high-risk MM according to Intergroup Francophone du Myeloma (IFM) stratification of the myeloma model, a higher incidence of high-risk genetic abnormalities was observed in patients with del(12p13) (62.1% vs. 30.4%, *P* = 0.001).

**Table 2:** Clinical and biological features of newly diagnosed MM patients associated with del(12p13)

Characteristics	Del(12p) *N* = 29	Without del(12p) *N* = 246	*P-value*
Durie-Salmon stage			0.245
I–II	2/29 (6.9)	35/236 (14.8)	
III	27/29 (93.1)	201/236 (85.2)	
ISS stage			0.006
I–II	8/29 (27.6)	128/234 (54.7)	
III	21/29 (72.4)	106/234 (45.3)	
BM plasmacytosis			0.034
≥ 50%	20/29 (69.0)	113/235 (48.1)	
< 50%	9/29 (31.0)	122/235 (51.9)	
LDH, U/L			0.669
≥ 220	6/28 (21.4)	40/221 (18.1)	
< 220	22/28 (78.6)	181/221 (81.9)	
Renal lesion	10/29 (34.4)	45/246 (18.3)	0.039
Bone lesions	24/29 (82.8)	180/246 (73.2)	0.264
Cytogenetic abnormality			
Del(13q)	16/29 (55.2)	103/246 (41.9)	0.171
Del(17p)	11/28 (39.3)	20/227 (8.8)	< 0.001
IGH translocation	21/28 (75.0)	119/215 (55.3)	0.048
t(11;14)	5/26 (19.2)	31/189 (16.4)	0.717
t(4;14)	10/26 (38.5)	35/189 (18.5)	0.019
t(14;16)	0/26 (0.0)	9/189 (4.8)	0.256
Amp(1q21)	11/29 (37.9)	100/201 (49.8)	0.234
High-risk [(any t(4;14), t(14;16) or del(17p)]	18/29 (62.1)	69/227 (30.4)	0.001

### Prognostic value of 12p13 deletion in newly diagnosed MM patients

**Figure 2:**
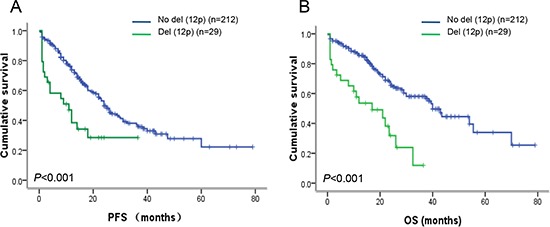
Survival analysis in newly diagnosed MM patients **(A)** Patients with del(12p13) had obviously inferior PFS (*P* < 0.001) when compared to patients without del(12p13). **(B)** Patients with del(12p13) had obviously inferior OS (*P* < 0.001) when compared to patients without del(12p13).

Follow-up data from 241 patients were analyzed to ascertain the prognostic value of 12p13 deletion in newly diagnosed MM patients. Totally 34 patients did not complete the follow-up examination due to poor contact information. The results showed that patients with del(12p) had significantly shortened PFS (11.0 vs. 24.0 months, *P* < 0.001) and OS (17.0 vs. 40.0 months, *P* < 0.001) than the patients without del(12p) (Figure 2A and 2B). In addition, we also investigated the impact of 12p13 gain on survival; however, no prognosis significance of 12p13 gain was observed in the present study. We further analyzed other risk factors that might affect the prognosis of this series of patients. Based on the univariate analysis shown in Table 3, patients with ISS stage III, LDH higher than 220 U/L, del(13q), del(17p), t(4;14), amp(1q21) and high-risk genetic abnormality had inferior survival than the control group. In multivariate analysis, when adjusted to the above prognostic variables, del(12p13) remained the powerful independent adverse factor for PFS (HR 2.29, 95% CI: 1.25–4.18, *P* = 0.007) and OS (HR 2.11, 95% CI: 1.07–4.17, *P* = 0.032). The other two independent factors were del(17p) (PFS: HR 2.50, 95% CI: 1.42–4.38, *P* = 0.001; OS: HR 1.90, 95% CI: 0.99–3.68, *P* = 0.050) and amp(1q21) (PFS: HR 2.67, 95% CI: 1.68–4.24, *P* < 0.001; OS: HR 1.91, 95% CI: 1.13–3.24, *P* = 0.016) (Table 4).

**Table 3:** Univariate analysis of risk factors for PFS and OS in newly diagnosed MM patients

Prognostic parameters	Median PFS (months)	*P* value	Median OS (months)	*P* value
ISS stage		0.013		0.002
I–II	26.0		55.5	
III	18.0		26.0	
LDH (U/L)		0.004		< 0.001
≥ 220	14.0		19.0	
< 220	24.0		40.0	
Del(13q)		0.012		0.025
Positive	19.0		32.0	
Negtive	29.5		Not reached	
Del(17p)		< 0.001		0.002
Positive	11.0		21.0	
Negative	24.0		54.0	
T(4;14)		0.022		0.043
Positive	15.5		24.0	
Negative	31.0		54.0	
Del(12p)		< 0.001		< 0.001
Positive	11.0		17.0	
Negative	24.0		40.0	
Amp (1q21)		< 0.001		< 0.001
Positive	15.5		23.5	
Negative	36.0		43.5	
Cytogenetic abnormality		0.002		0.005
High-risk	17.0		23.5	
Non high-risk	26.0		43.5	

Cytogenetic high-risk: any t(4;14), t(14;16) or del(17p)

**Table 4:** Multivariate analysis of risk factors for PFS and OS in newly diagnosed MM patients

Prognostic parameter	HR for PFS (95% CI)	*P* value	HR for OS (95% CI)	*P* value
ISS stage III	1.11 (0.72–1.71)	0.637	1.19 (0.70–2.03)	0.514
LDH	1.24 (0.73–2.10)	0.420	1.49 (0.82–2.71)	1.490
Del(13q)	1.15 (0.73–1.80)	0.550	1.32 (0.76–2.28)	0.322
Del(17p)	2.50 (1.42–4.38)	0.001	1.90 (0.99–3.68)	0.050
t (4;14)	1.21 (0.69–2.13)	0.507	1.09 (0.56–2.14)	0.794
Del(12p)	2.29 (1.25–4.18)	0.007	2.11 (1.07–4.17)	0.032
Amp (1q21)	2.67 (1.68–4.24)	< 0.001	1.91 (1.13–3.24)	0.016

### Del(12p) combined with high β2-MG, high LDH or bone lesion can further predict the high-risk subgroups in newly diagnosed MM patients

**Figure 3:**
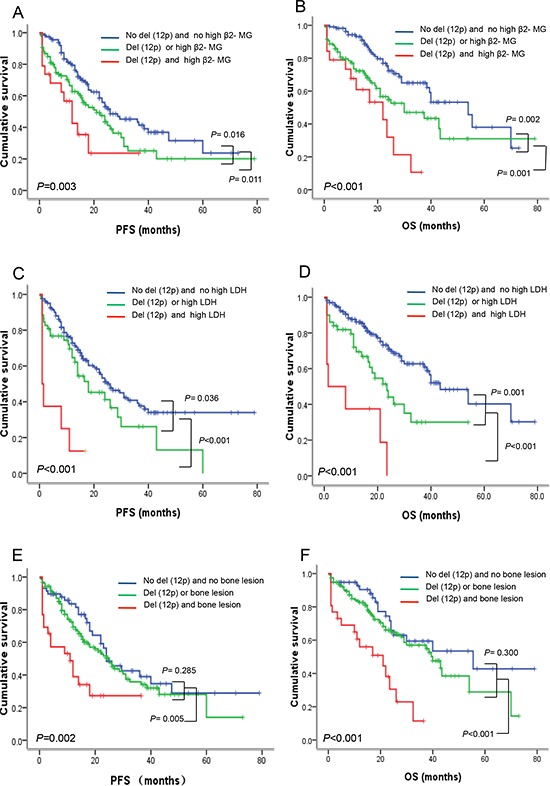
Del(12p13) combined with high β2-MG, high LDH and bone lesion can further divide patients into subpopulations with different risk features **(A–B)** Del(12p13) combined with β2-MG can divide patients into three subpopulations with different PFS (*P* = 0.003) and OS (*P* < 0.001). Patients with del(12p) and high β2-MG simultaneously had the shortest PFS and OS, followed by patients with del(12p) or high β2-MG, patients without del(12p) and high β2-MG had relatively better outcome. **(C–D)** Del(12p13) combined with LDH can also divide patients into three subpopulations with different PFS (*P* < 0.001) and OS (*P* < 0.001). Patients with del(12p) and high LDH simultaneously had the shortest PFS and OS, followed by patients with del(12p) or high LDH, patients without del(12p) and high LDH had relatively better outcome. **(E–F)** Patients with del(12p) and bone lesion simultaneously had shorter PFS and OS than patients without del(12p) and/or bone lesion.

We investigated whether del(12p) combined with others factors could further identify the high-risk subgroups. It is surprising that del(12p) combined with high β2-MG, high LDH or bone lesion was a good predictor marker for dividing patients into subpopulations with different OS time (Table 5). Patients with del(12p) and high β2-MG had inferior outcome for PFS (12.0 vs. 21.0 vs. 26.0 months, *P* = 0.003) and OS (22.0 vs. 30.0 vs. 54.0 months, *P* < 0.001) than patients with del(12p) or high β2-MG and patients without del(12p) and high β2-MG (Figure 3A and 3B). A similar analysis for the prediction of OS was also performed on the parameters of high LDH and bone lesion (Figure 3C–3F). In addition, patients with del(12p) and high LDH at the same time had a very aggressive clinical course with the OS time from 1.0 month to 23.5 months. Among 8 patients, 5 patients received bortezomib-based chemotherapy and 3 patients received thalidomide-based chemotherapy; however, possibly due to the limited number and short follow-up time in these patients, the median estimated PFS and OS for patients with del(12p) and high LDH at the same time were only 1.0 and 1.5 months.

**Table 5:** Del(12p) combined with high β2-MG, high LDH or bone lesion can further identify the high-risk subgroups in newly diagnosed MM patients

Subgroups	Median PFS (months)	*P* value	Median OS (months)	*P* value
**Del(12p) and β2-MG**		0.003		< 0.001
No del(12p) and no high β2-MG (*n* = 110)	26.0		54.0	
Del(12p) or high β2-MG (*n* = 106)	21.0		30.0	
Del(12p) and high β2-MG (*n* = 19)	12.0		22.0	
**Del(12p) and LDH**		< 0.001		< 0.001
No del(12p) and no high LDH (*n* = 162)	26.0		43.5	
Del(12p) or high LDH (*n* = 50)	18.0		23.5	
Del(12p) and high LDH (*n* = 8)	1.0		1.5	
**Del(12p) and bone lesion**		0.002		< 0.001
No del(12p) and no bone lesion (*n* = 58)	24.0		55.5	
Del(12p) or bone lesion (*n* = 157)	25.0		40.0	
Del(12p) and bone lesion (*n* = 26)	11.0		21.0	

### Bortezomib did not significantly improve the survival of patients with del(12p)

**Figure 4:**
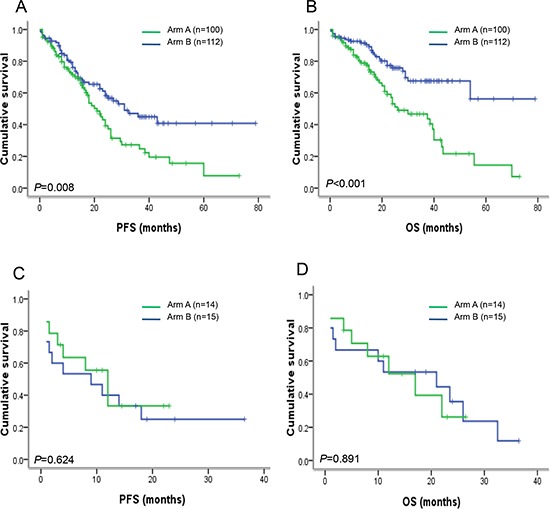
PFS and OS in newly diagnosed MM patients with or without del(12p13) after receiving thalidomide-based or bortezomib-based therapy **(A–B)** In patients without del(12p13), PFS (*P* = 0.008) and OS (*P* < 0.001) were obviously improved after receiving bortezomib-based therapy compared to patients receiving thalidomide-based therapy. **(C–D)** However, in patients with del(12p13), patients with thalidomide-based and bortezomib-based therapy had similar PFS (*P* = 0.624) and OS (*P* = 0.891), suggesting that bortezomib-based therapy could not improve the poor survival of patients with del(12p13).

In thalidomide-based chemotherapy group (arm A), the median PFS and OS of patients with del(12p) were 12.0 vs. 21.0 months (*P* = 0.059) and 17.0 vs. 26.0 months (*P* = 0.026) when compared to patients without del(12p). In bortezomib-based chemotherapy group (arm B), the median PFS and OS of patients with del(12p) were 9.0 vs. 31.0 months (*P* = 0.001) and 21 vs. not reached (*P* < 0.001) when compared to patients without del(12p). In addition, in patients without del(12p), the median PFS and OS of patients treated with bortezomib-based chemotherapy were 31.0 vs. 21.0 months (*P* = 0.008) and not reached vs. 26.0 months (*P* < 0.001) when compared to patients treated with thalidomide-based chemotherapy (Figure 4A and 4B). However, in patients with del(12p), no statistically significant difference between bortezomib-based and thalidomide-based chemotherapy groups was observed (PFS: *P* = 0.624; OS: *P* = 0.891) (Figure 4C and 4D). It suggests that bortezomib-based chemotherapy could obviously improve the survival of patients without del(12p), but not overcome the negative impact of del(12p).

## DISCUSSION

The deletion of chromosome 12p was reported as a novel independent poor prognostic predictor in 192 newly diagnosed patients using SNP-based array in 2009 and has garnered increased attention in recent years [5, 7, 8]. Although the result is subsequently validated in a cohort of 273 patients with myeloma [14], two studies are failed to confirm this using a FISH probe covering the similar chromosome region [9, 10]. So, the prognostic value of del(12p) in MM patients remains controversial and routine testing for del(12p) has not currently accepted by the International Myeloma Working Group (IMWG) [3]. This study has focused on the status of 12p abnormality in plasma cell dyscrasias and comprehensively evaluated the prognostic value of del(12p) in newly diagnosed MM patients in the background of the novel agent-based therapy.

According to our current knowledge, the abnormality of 12p13 in myeloma patients by FISH has been only mentioned in several studies. The prevalence of 12p deletion (10.5%) in our cohort is consistent with previous reports with 13%, 8% and 11% of 12p deletion, respectively [5, 9, 10]. In addition, the higher deletion rate of 12p is observed in relapsed (14.4% vs.10.5%, *P* = 0.314) and sPCL (37.5% vs. 10.5%, *P* = 0.008) patients than newly diagnosed patients. However, no patients with MGUS have 12p deletion. Interestingly, we have also found that 4.4% of newly diagnosed and 12.2% of relapsed patients have 12p gain (*P* = 0.007). The mechanism of higher incidence of 12p disorders in the advanced stage of MM and sPCL remains unclear, which is possibly due to the chromosomal instability in the progression of plasma cell dyscrasias.

We have further explored the correlation between del(12p) and other clinical parameters in newly diagnosed MM patients. To our surprise, del(12p) is significantly associated with high tumor burden-related indicators including ISS III, BM plasmacytosis higher than 50%, and renal lesion. β2-MG, a good marker reflected tumor burden, is defined as ISS III at the level of larger than 5.5 mg/dL; however, the level of β2-MG is usually influenced by renal lesion. Thus, we further analyzed the correlation between del(12p) and β2-MG excluding patients with β2-MG higher than 5.5 mg/dL and renal lesion simultaneously, the strong correlation remains existence (*P* = 0.025). In addition, we have also found that cases with 12p deletion have a obviously higher incidence of high-risk genetic aberrations including del(17p) and t(4;14). In contrast, Jiang et al. have detected the 12p deletion in 88 newly diagnosed MM patients, and do not observe the association between 12p deletion and 13q deletion or t(4;14), but their results also support our finding that MM and sPCL cases with 12p deletion tended to have more frequency of del(17p) when compared to patients without del(12p) (42.0% vs.11% in MM and 75.0% vs. 50.0% in sPCL, respectively) [9]. The discrepancy is possibly due to the small size of the samples in Jiang's investigation. Our results have demonstrated that 12p deletion is a secondary event rather than a primary event associated with disease progression and high tumor burden in MM.

The prognostic impact of 12p deletion on MM patients in several different cohorts remains inconsistent. Avet-Loiseau et al. have identified the loss of 12p as an independent poor prognostic factor for PFS and OS in newly diagnosed MM using SNP-based array (192 cases) and FISH (273 cases) [5]. However, an analysis of 256 newly diagnosed MM patients from the Medical Research Council (MRC) Myeloma IX trial presents no statistically significant difference (*P* = 0.061), although a trend for shorter overall OS time in patients with del(12p13) (3.33 vs. 4.18 years) is observed [10]. Furthermore, results from Jiang's investigation have described that patients with and without del(12p) have similar PFS (median 23.5 vs. 21.9 months, *P* = 0.616) and OS (median 44.5 vs. 91.4 months, *P* = 0.105) [9]. Our results are comparable with the report from Avet-Loiseau, and have demonstrated that patients with del(12p13) have remarkably shorter PFS (11.0 vs. 24.0 months, *P* < 0.001) and OS (17.0 vs. 40.0 months, *P* < 0.001) than patients without del(12p13). In multivariate analysis, del(12p13) still remains its independent prognostic significance for PFS (HR 2.29, 95% CI: 1.25–4.18, *P* = 0.007) and OS (HR 2.11, 95% CI: 1.07–4.17, *P* = 0.032). Strikingly, as the prognostic model including del(12p), β2-MG and amp(5q31.3) suggested from Avet-Loiseau's investigation, we also have found that del(12p) combined with high β2-MG, high LDH or bone lesion is a good predictor marker for dividing patients into subpopulations with different OS time, especially, the patients with del(12p) and high LDH at the same time have a very aggressive clinical course and high mortality rate. These results strongly support that del(12p) is a powerful prognostic marker for defining subpopulation with high-risk features.

Finally, our data have shown that the negative outcome of del(12p) could not be obviously improved by bortezomib-based therapy, suggesting that del(12p) may mediate the resistance to bortezomib-based therapy. Zhan et al. have first disclosed that *CD27* located in 12p13.31 is one of the most significantly under-expressed genes in MM [11]. Davies et al. have subsequently conformed that the down-regulation of *CD27* is linked to process from normal through MGUS to MM [15]. Moreau et al. have evaluated CD27 expression on normal plasma cells (PCs), PCs from MM patients at diagnosis or relapse and human myeloma cell lines (HMCL) using flow cytometry. The results have revealed that CD27 is expressed by normal PCs, but is lacking in PCs from MM patients at diagnosis (36%), relapse (47%) and HMCL (92%), and patients with CD27 negative have poor survival [16]. All of these results support that *CD27* gene may play an important role in MM progression. As a member of the TNFR superfamily, CD27 provides co-stimulatory signals for the proliferation of T- and B-cells and immunoglobulin production and apoptosis of B-cells [17]. Down-regulation of *CD27* gene in MM may prevent apoptotic program mediated by interleukin 10 (IL-10) [18]. Moreover, *CD27* activates both the classical and alternative nuclear factor-**κ**B pathways and the under-expression of *CD27* in CD-1 myeloma subgroup is reported to be associated with a lower NF-**κ**B signature when compared to CD-2 myeloma subgroup [19, 20]. These results could explain that bortezomib-based therapy targeting the NF-**κ**B pathway may not overcome the negative impact on patients with del(12p), and further studies are necessary to disclose the underlying mechanisms.

In conclusion, del(12p) is heterogeneous and associated with high tumor burden in myeloma. Patients with del(12p13) had adverse prognosis and were resistant to bortezomib-based therapy. Del(12p13) combined with other markers including high β2-MG, high LDH, and bone lesion can further identify patients with high-risk features. So, we suggest all newly diagnosed MM patients should be routinely detected 12p deletion by FISH to evaluate the prognosis.

## METHODS

### Patients and samples

All newly diagnosed MM patients were derived from a prospective, non-randomized clinical trial (BDH 2008/02). The trial was approved by the ethic committee of the Institute of Hematology, Chinese Academy of Medical Sciences, and Peking Union Medical College, according to the guidelines of the 1996 Helsinki Declaration. Patients were homogeneously provided with thalidomide-based (arm A) or bortezomib-based (arm B) treatment according to our previous report [21]. Arm A consisted of TAD (thalidomide, adriamycin and dexamethasone) or TCD (thalidomide, cyclophosphamide and dexamethasone); Arm B consisted of BCD (bortezomib, cyclophosphamide and dexamethasone) or PAD (bortezomib, adriamycin and dexamethasone). After at least four cycles of treatments with partial remission or better efficacy, patients underwent consolidation therapy with the patient's original regimen. Subsequently, patients were treated with thalidomide (100–150 mg/d) for one year to maintain response.

### Fluorescence in situ hybridization

Mononuclear cells from patients with MM were separated by gradient density centrifugation (Ficoll-Hypaque; Eurobio, Les Ulis, France). Plasma cells were then purified using CD138-coated magnetic beads according to the manufacturer's instructions (Miltenyi Biotec, Germany), thus enabling plasma cell purity higher than 90%. Plasma cells were then assessed using DNA probes specific for the following chromosomal aberrations: del(13q14), del(17p), amp(1q21), IgH translocation, t(11;14), t(4;14), and t(14;16). Del(12p) was assessed using a bacterial artificial chromosome (BAC) probe specific for 12p13.31 region covered *CD27* gene (RP11–72G18). FISH analysis was performed as previous report [21]. For each probe, 200 plasma cells were scored and the cut-off level was set at 20% for both deletion and amplification according to the recommendation of European Myeloma Network (EMN) [22].

### Statistical analysis

Statistical analysis was conducted using SPSS version 19.0 software. Progression-free survival (PFS) was calculated from the date of diagnosis until disease progression or death, and overall survival (OS) was calculated from the date of diagnosis until death. Comparison of categorical variables was conducted by chi-square test and non-parametric test. Survival curves were plotted using the Kaplan-Meier method. Differences between curves were tested for statistical significance using the log-rank test. Multivariate analysis of variables associated with survival was conducted by Cox Proportional-Hazard model for both PFS and OS. A statistically significant difference was considered at*P* ≤ 0.05.
